# Study on the germination rate of maize seeds based on improved YOLOv8n model

**DOI:** 10.3389/fpls.2025.1555440

**Published:** 2025-05-26

**Authors:** Helong Yu, Jiayao Zhao, Chunguang Bi, Jing Chen, Ming Zhao

**Affiliations:** ^1^ College of Information Technology, Jilin Agricultural University, Changchun, China; ^2^ Smart Agriculture Research Institute, Jilin Agricultural University, Changchun, China; ^3^ Jilin Province Zhongnong Sunshine Data Co., Ltd, Changchun, China

**Keywords:** YOLOv8n, corn seed, germination trend detection, digital agriculture, detection model

## Abstract

The germination potential of corn seeds, a key index for assessing their quality and directly associated with the ultimate corn yield, is currently defined in a way that cannot effectively portray the seed germination rate, and the prevalent measurement methods are traditional, consuming substantial process resources. To tackle these issues, this paper employs a public corn seed germination dataset, adds noise to it to simulate real - world production conditions, and ultimately acquires a dataset comprising 8148 images. It then proposes an enhanced YOLOv8 target detection model, EBS - YOLOv8, for detecting corn seed germination. Specifically, the ECA lightweight attention mechanism is introduced to decrease small - target feature loss, assist in accurate target recognition, and remove redundant features; simultaneously, the P2BiFPN multiscale feature fusion technique is utilized to boost the detection ability for small targets; furthermore, the ScConv convolution is adopted to enhance the feature - extraction capacity and improve detection accuracy. Combined with the improved model, this paper also proposed a mathematical modeling algorithmnew method for measuring seed germination potential and observing seed germination rate. The results indicate that the proposed model attains a mean average precision at 50% Intersection over Union (mAP50) value of 98.9%, a mean average precision in the range of 50% - 95% Intersection over Union (mAP50 - 95) value of 95.8%, an accuracy of 96.7%, and a recall of 96.3%. In comparison with the original model, the mAP50 has increased by 0.9% and the mAP50 - 95 value has witnessed a 3.7% increment. The experiments have demonstrated that the research method for germination potential put forward in this paper can effectively depict the rate variation of seeds during the germination process, thus offering a novel perspective for future research on seed germination potential.

## 1 Introduction

Corn is one of the most important food crops in the world, widely grown all over the world, i.e. it is the main source of food for human beings and is also widely used in animal feed and industrial raw materials. The quality of corn seed is directly related to the yield of corn, and seed germination rate and germination potential an important indexes for evaluating the quality of corn seed, which is directly related to the germination ability of the seed and the beginning of growth and development (seed germination rate refers to the percentage of the seed that germinates and forms a normal seedling under certain conditions. Germination potential, on the other hand, refers to the speed and intensity of seed germination in a certain period, which is one of the manifestations of seed vitality), it can be said that the two are based on germination rate, while the concept of germination potential is further extended from the basis of germination rate. Seed germination status testing is important in the process of plant growth (Yuting and Yuji, 2021; Šerá and Hnilička, 2023), and an in-depth understanding of the germination rate and germination potential of maize seeds is an important guide for growers to select high-quality seeds, increase yields, and improve the quality of their products (Yang et al., 2017; He et al., 2022; Divya Venkata et al., 2023; Zhang et al., 2023).

Seeds with high germination rates show rapid germination in the field and possess greater resistance to adversity; on the contrary, seeds with low germination rates usually germinate slowly in the field, emerge irregularly, and are susceptible to the growing environment, which may lead to lower yields of agricultural products (Reed et al., 2022). Traditional methods of seed germination detection usually rely on experienced personnel who mark seed categories by observing seed radicle and germ length (Yang et al., 2013). In this process, germination detection is carried out by human observation and counting to determine the number of seeds that have germinated within 7 days. However, this method requires a high level of experience on the part of the inspector, and the process of repeating the germination rate test is cumbersome, time-consuming, and laborious, and is prone to introduce subjective errors, resulting in inconsistent and poorly reproducible results between different inspectors. With the development of smart agriculture, germination tests are gradually transforming into intelligence (Zhang et al., 2017; Zhang et al., 2018; Party Satisfaction et al., 2020). However, traditional testing methods rely heavily on finely controlled conditions, which are difficult to realize. The strict experimental conditions and long testing time (usually 7 days) as well as many methods using chemical measurements may cause potential damage to the seeds, resulting in non-reusability (Filho, 2015). Therefore, the limitations of traditional germination rate and germination potential assays highlight the urgency of developing a rapid, nondestructive, and accurate assay that reduces the cost of the assay while increasing the speed and accuracy of the assay.

In recent years, deep learning has developed rapidly and has been widely used in agriculture, especially in seed germination detection, and many researchers have begun to explore its potential (Party Satisfaction et al., 2020; Zhang et al., 2021). Joosen et al. (2010) proposed a semi-automatic method to design a germination instrument that determines whether a seed has germinated or not using a high-throughput score. The instrument can handle many samples that may germinate under different environmental conditions. However, the method requires good contrast between the radicle and the seed coat, a requirement that may limit its application in some crops. Zhao et al. (2022) utilized techniques such as image segmentation, transform encoder, small target detection layer, and CDIOU loss to improve the accuracy of detection. They developed a convolutional neural network (YOLO-r) that can effectively detect the germination status of rice seeds and automatically evaluate the total number of germination with an average accuracy of 0.9539 and an average absolute error of predicting germination rate mainly within 0.1. Bai et al. (2023) constructed a seed germination discrimination model named DB-YOLOv5 by combining machine vision technology and deep learning methods to rapidly detect the germination rate, germination potential, germination index, and average germination days of wheat seeds, and verified in experiments that the accuracy of the model for wheat seed germination discrimination was as high as 98.5%. Zheng et al. (Yao et al., 2023) designed a semi-automatic germinator through the YOLO algorithm, which successfully realized the detection of the germination rate of rice seeds in the field and assessed the seed germination rate through image analysis. Liu et al. (2023) tried to improve the local linear embedding with different distance metrics and proposed a fast detection method for corn seed germination based on improved local linear embedding and near-infrared spectroscopy. Aiming at the physiological and physical differences of rice seeds at different aging times, Fang et al. (2016) proposed a fast and nondestructive detection method for rice seed germination based on infrared thermography and a generalized regression neural network. Mark et al. (Iradukunda et al., 2024) explored cost-effective imaging techniques for rapid assessment of seedling vigor, providing practical solutions to common problems in agricultural research. Zhang et al. (2025) proposed an improved safflower detection model named WED-YOLO based on YOLOv8n, which enables accurate identification of safflowers in complex environments and has made outstanding contributions to the automated harvesting of safflowers. All indicators of the new model have been improved compared with those of the baseline model. In order to accurately detect safflower filaments under different lighting conditions, with foliage obstruction and various weather conditions, Shi Ruiming et al (Zhang et al., 2023) proposed an improved Fast R-CNN filament model. This model enables accurate and rapid identification of safflower filaments on sunny, cloudy, and overcast days, as well as under conditions of sunlight, backlight, foliage obstruction, and dense occlusion. It provides technical support for the identification of small-scale crops. Although the above studies achieved good accuracy in germination rate detection, their definitions of germination potential are still relatively simple and based mainly on the consideration of germination time, while failing to delve into more complex metrics such as germination rate and germination germination trend.

To address the shortcomings of existing studies in the detection of germination rate and germination potential of maize seeds, this paper proposes an improved model based on YOLOv8n for fast, accurate, and non-destructive detection. First, the diversity of the dataset was enhanced by data enhancement techniques to enhance the learning ability of the model. Second, the structure of the model was innovated by adding three new modules to improve the accuracy of detecting small target corn seeds while effectively controlling the number of parameters of the model. To further analyze the characteristics of germinating seeds, this paper uses traditional algorithms to measure the germinating seed portion of the image, combines statistical methods to process the data, and fits regression equations through mathematical modeling methods to depict the trend of seed germination. Finally, the equation was derived and the curve obtained was able to effectively represent the germination potential of corn seeds within a specified period. The experimental results show that the model has high robustness and generalization performance in detecting maize seed germination, possessing the potential for practical application in mobile applications, and the proposed method of fitting the equation provides a new idea for accurately depicting the seed germination potential. The contributions are summarized as follows:

(1) Dataset construction: A specialized dataset for corn seed germination detection was constructed using data enhancement based on the existing public dataset to improve the diversity of the data;
(2) Lightweight Attention Mechanism: A lightweight ECA attention mechanism is introduced to discard redundant features such as noise - interfering points in the background of seed images, focus on effective features related to the germination rate, and simultaneously avoid increasing the number of model parameters;
(3) Small-target detection and feature fusion: adding a small-target detection layer and BiFPN structure to increase the multi-scale feature fusion capability and improve the detection accuracy of small-target corn seeds;
(4) Improvement of convolutional layer: add a layer of ScConv convolution to increase the depth of the network and strengthen the feature extraction ability, and at the same time reduce the redundant parameters in the upper layer, to improve the overall performance of the model;
(5) Redefinition of germination potential: by combining deep learning techniques with mathematical modeling, germination potential has been redefined to depict the germination potential of seeds.

## 2 Materials and methods

### 2.1 Dataset construction

#### 2.1.1 Image acquisition

**Figure 1:**
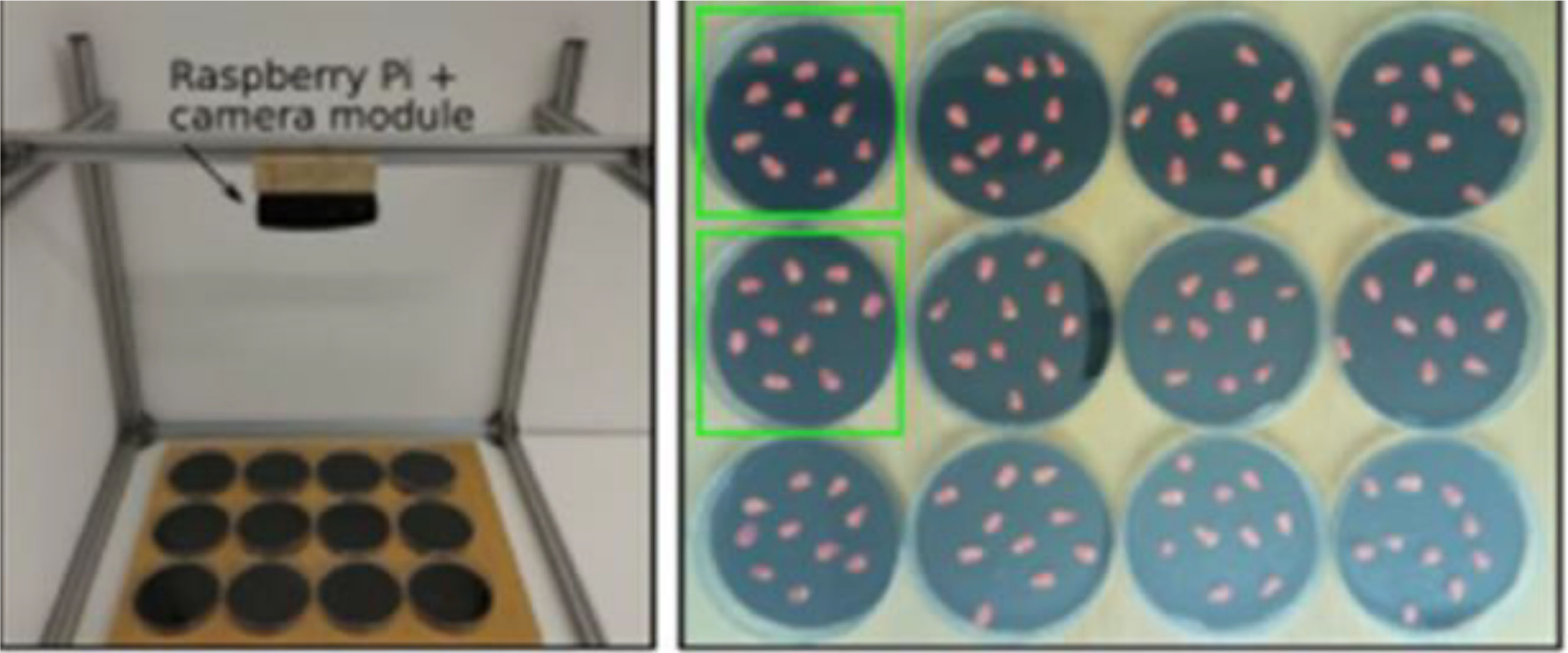
Image acquisition device.

**Figure 2:**
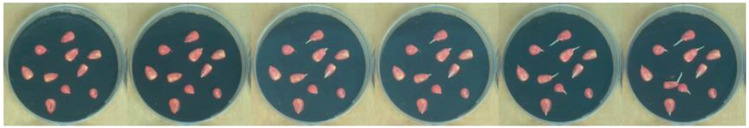
Seed germination images.

This experiment utilizes a publicly accessible dataset, which employs digital imaging techniques to capture images of seeds in three distinct germination states. In the experimental setup, seeds were carefully placed in Petri dishes and then positioned on a black cloth. This was done to guarantee a high contrast between the emerging radicle and the background, facilitating clear image capture.During the experiment, the seeds were irrigated with tap water. To minimize water evaporation, the Petri dish was covered with a lid, as depicted in **Figure 1**. All images were captured under the same artificial light source over a period of 48 hours, with intervals of 30 minutes between each shot. This systematic approach ensured comprehensive documentation of the entire germination process.The dataset used in this experiment can be accessed at ‘http://dx.doi.org/10.17332/4wkt6thgp6.2’. In this study, the part of the maize seeds in the dataset was used. There are a total of 84 culture dishes in this part, and a total of 8,148 images were taken, covering the entire germination process of maize seeds from the initial stage to 48 hours, as shown in **Figure 2**.

#### 2.1.2 Data augmentation

**Figure 3:**
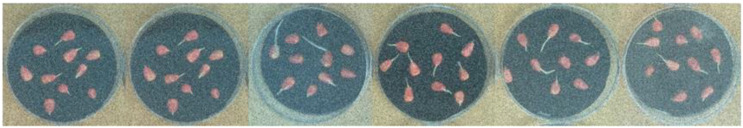
Seed germination image after adding noise.

In this study, the original dataset is expanded using image enhancement techniques. Adding noise is a commonly used method for image enhancement, such as Gaussian noise, salt and pepper noise, Poisson noise, and uniform noise. The purpose in this paper is to increase the richness and interference of the images, and further improve the detection performance of the model. Poisson noise is more suitable for medical imaging, and the interference effect added by uniform noise has relatively low randomness. Gaussian noise and salt and pepper noise perform better in terms of the degree of interference. However, the principle of salt and pepper noise is to change some pixel points to white or black, which is obviously more suitable for the actual situation of corn seed germination and conforms to our research. Therefore, salt and pepper noise is chosen for data augmentation. A portion of the data from the 8,148 original images underwent processing by applying the sp_noise function, with the noise factor configured as 0.2. Subsequently, the dataset was partitioned into a training set, a test set, and a validation set according to a ratio of 7:2:1. Post - processing, a total of 5,703 training - set images, 1,630 test - set images, and 815 validation - set images were ultimately acquired, as illustrated in **Figure 3**. The enhanced dataset is only used for model training to improve the model’s comprehensive performance and enhance its detection ability. In the subsequent experiments on germination rate and germination potential, we used the original dataset without noise enhancement. The reason for this is that the focus of this part of the research is to evaluate the germination potential of seeds, which requires high precision. Using the model trained with the enhanced dataset to identify the original image data can better assist us in our research.

### 2.2 Selection of models

Deep - learning - based target detection networks play a vital role in detecting seed germination. At present, numerous networks exhibit excellent performance in the field of target detection, including RCNN, SSD, CenterNet, and the YOLO series. The YOLO series of algorithms adopts an end - to - end approach. This not only remarkably enhances the detection speed but also allows for the direct acquisition of the target’s positional coordinates and classification labels, which is of great significance for our subsequent research on germination potential.YOLOv8, as a more recent iteration of the YOLO series of algorithms, demonstrates a substantial improvement in performance compared to its predecessors. Its advantages such as easy deployment, rapid detection, and high accuracy rate make it a crucial tool for detecting corn seed germination. In this paper, YOLOv8n is compared with Rcnn, SSD, and CenterNet - ResNet50, as presented in **Table 1**.

**Table 1:** Model selection comparison.

Model	Precision(%)	Recall(%)	mAP50(%)	mAP50-95(%)	Params(M)
YOLOv8n	95.1	**94.0**	**98.0**	**92.1**	**3.00**
Faster Rcnn-ResNet50	92.9	91.6	89.9	55.5	137.10
SSD-VGG	93.6	89.9	90.1	81.9	26.29
CenterNet-ResNet50	**96.5**	90.8	92.6	88.7	32.67

Key metrics with outstanding performance are highlighted in bold.

### 2.3 EBS-YOLOv8 construction

**Figure 4:**
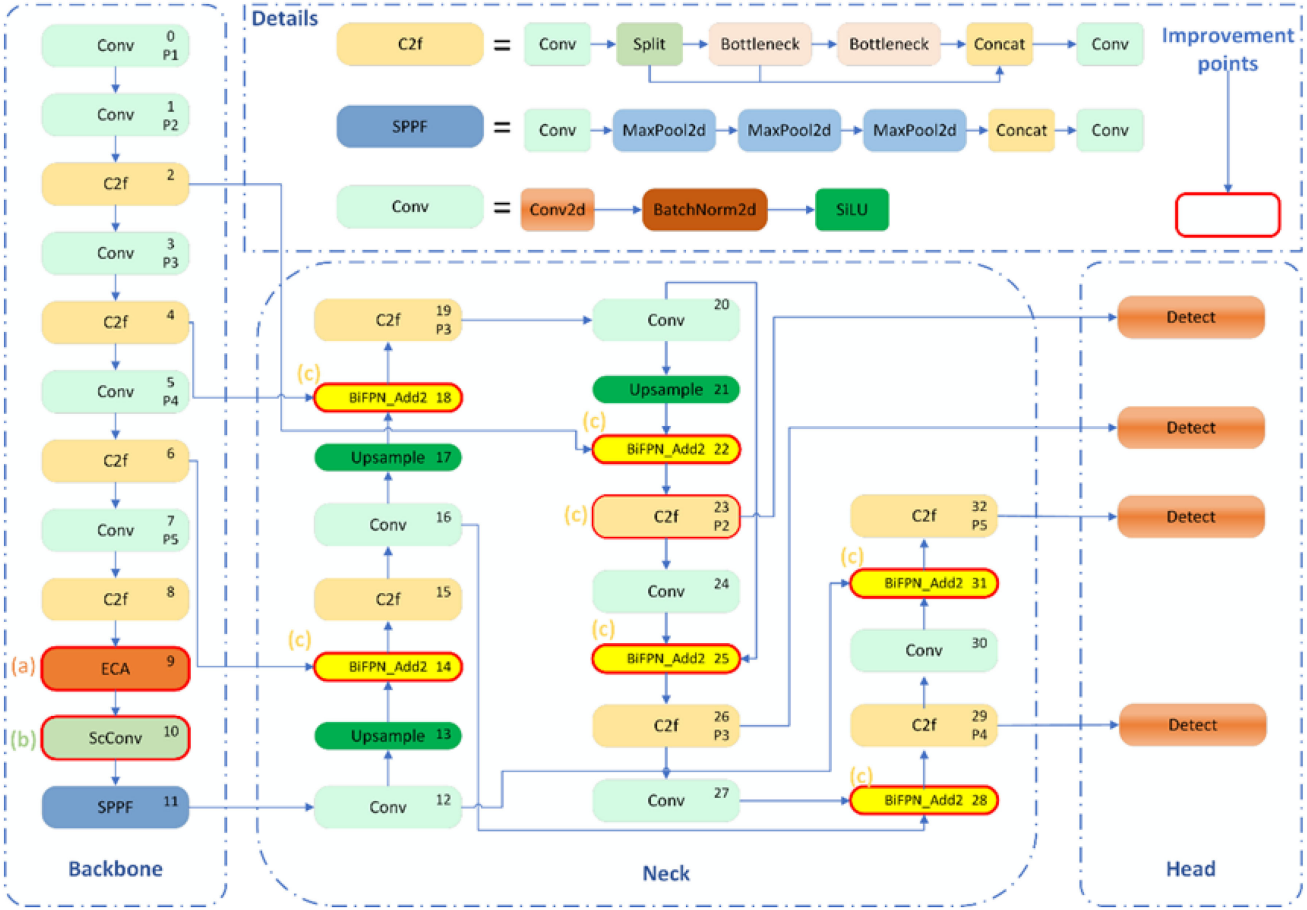
EBS-YOLOv8 network structure.

The YOLOv8n model consists of a Backbone, Neck, and Head. Among them, the Backbone serves as the backbone network, which mainly consists of Conv module and C2f(Cross Stage Partial - 2 with Fused Shortcut) and SPPF(Spatial Pyramid Pooling - Fast) modules for feature extraction; the Neck fuses features at different scales; and the Head network is used for final target prediction. The model performs well in the task of germination detection of corn seeds. To further improve its performance this paper improves YOLOv8n and names it EBS-YOLOv8 as shown in **Figure 4**. In Backbone, the ECA(Efficient Channel Attention) lightweight attention mechanism (a) is introduced to enhance the attention to useful information features during feature extraction to optimize resource allocation. This improvement not only enhances the model performance but also alleviates the conflict between performance and complexity, with a small number of additional parameters. The next layer of ECA incorporates the ScConv(Spatial and Channel Reconstruction Convolution) module (b), which utilizes spatial and channel redundancy in features for compression, reducing redundant computation and enhancing feature learning. In addition, a small target detection layer is added to the Neck network, and the traditional FPN(Feature Pyramid Network) is upgraded to a P2BiFPN(Bidirectional Feature Pyramid Network based on P2 layer) for efficient fusion of features to further improve the detection accuracy.

#### 2.3.1 ECA attention mechanism

In the dataset of germinated seeds with added noise, the characteristics of seeds in the early germination stage were not distinct. When detecting newly germinated seeds, the model faced challenges in accurately identifying whether they had germinated, particularly when noise points partially overlapped with the seed buds and the similarity between the noise and the buds was high.

**Figure 5:**
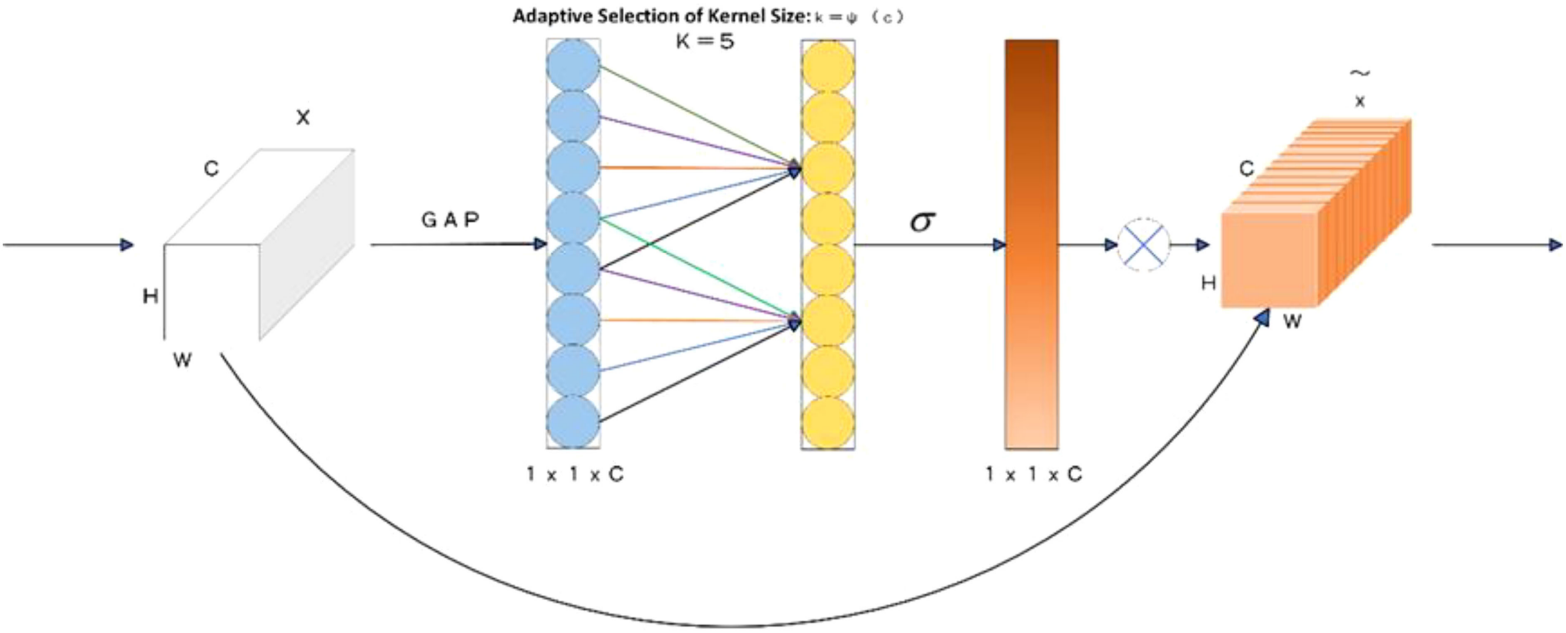
ECA attention mechanism.

To address this issue, eliminate redundant features, and prevent overfitting, this paper incorporates the ECA (Efficient Channel Attention) attention mechanism into the Backbone network, as depicted in **Figure 5**. The ECA attention mechanism processes the input feature maps through global average pooling. This operation transforms the feature maps from the initial [h, w, c] matrix into a [1, 1, c] vector. Subsequently, an adaptive 1D convolution kernel size is calculated based on the number of channels in the feature map. This kernel is then utilized for 1D convolution to obtain the weight values for each channel of the feature map. Finally, the normalized weights are multiplied channel - by - channel with the original input feature maps to generate weighted feature maps.

In this process, the ECA mechanism directly applies a 1x1 convolution after the global average pooling layer. By doing so, it circumvents the use of a fully connected layer, thereby preventing dimensionality reduction and effectively capturing cross - channel interaction information. This design significantly enhances the model’s detection performance while only slightly increasing the number of parameters.

#### 2.3.2 Fusion of small target layer and BiFPN structure

**Figure 6:**
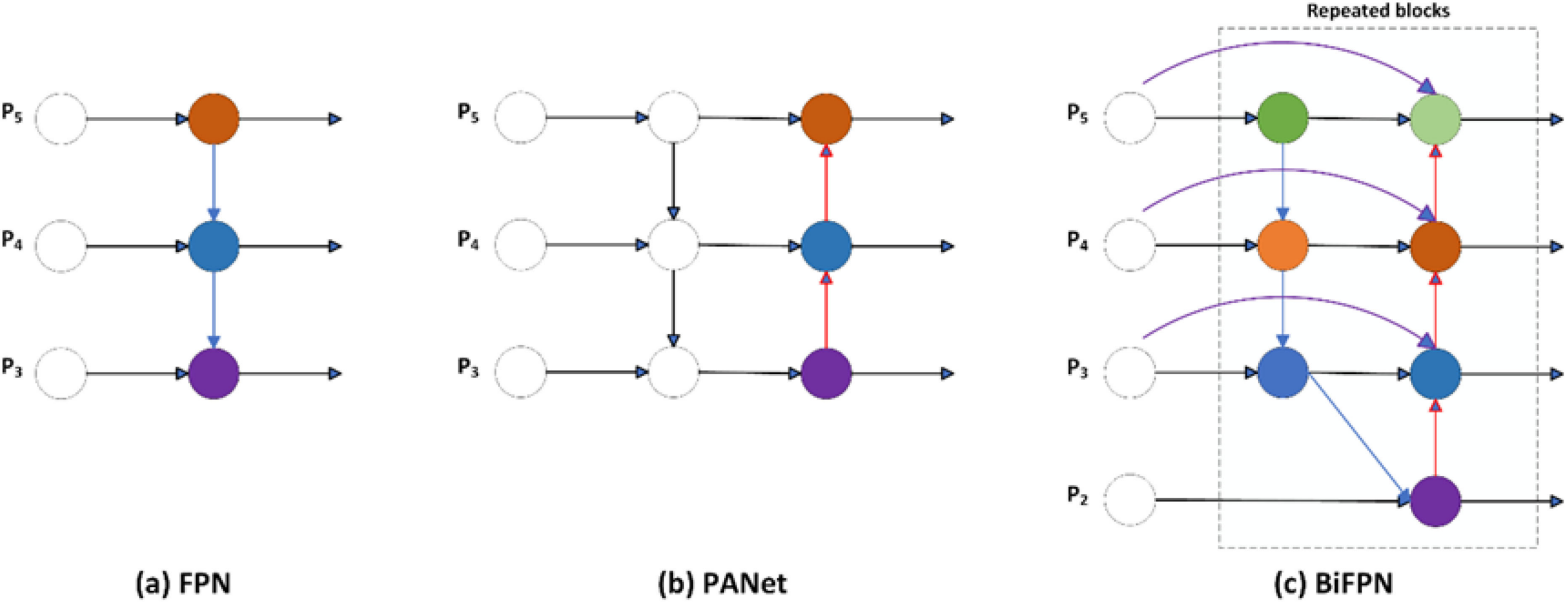
**(a)** conventional FPN structure, **(b)** PANet structure, **(c)** BiFPN structure.

**Figure 6a** illustrates the traditional Feature Pyramid Network (FPN) structure. It achieves multi - scale feature fusion of P3 - P7 by introducing a top - down path. Nevertheless, this method might result in less comprehensive utilization of feature information. Consequently, PANet incorporates a bottom - up path into the FPN, as presented in **Figure 6b**. Although this improvement enhances the information features to a certain extent, there is still room for further improvement.

In this research, a small - target detection layer P2 is introduced in EBS - YOLOv8. This enables the fusion of tiny features and their combination with Bi - FPN (Bidirectional Feature Pyramid Network). The Bi - FPN structure, depicted in **Figure 6c**, introduces bidirectional feature propagation, involving both top - down and bottom - up feature flows. This mechanism facilitates more comprehensive and rich information transfer and feature fusion among different layers.

Furthermore, Bi - FPN incorporates the operations of feature adjustment and feature selection. Feature tuning optimizes the feature weights to enhance the overall fusion results, thereby improving the fusion performance. Meanwhile, during the feature selection stage, useful features are dynamically selected based on different importance and confidence levels. This design not only enables higher accuracy in the detection process but also strikes a balance between efficiency and accuracy. It reduces the computational load while enhancing the model’s performance.

#### 2.3.3 Space and channel reconstruction convolution

Convolution holds an irreplaceable position and plays a pivotal role in deep - learning architectures. Appropriately augmenting the network depth can enhance its performance. However, when adding convolutional layers, it is essential to prevent resource waste caused by the extraction of redundant features.

**Figure 7:**
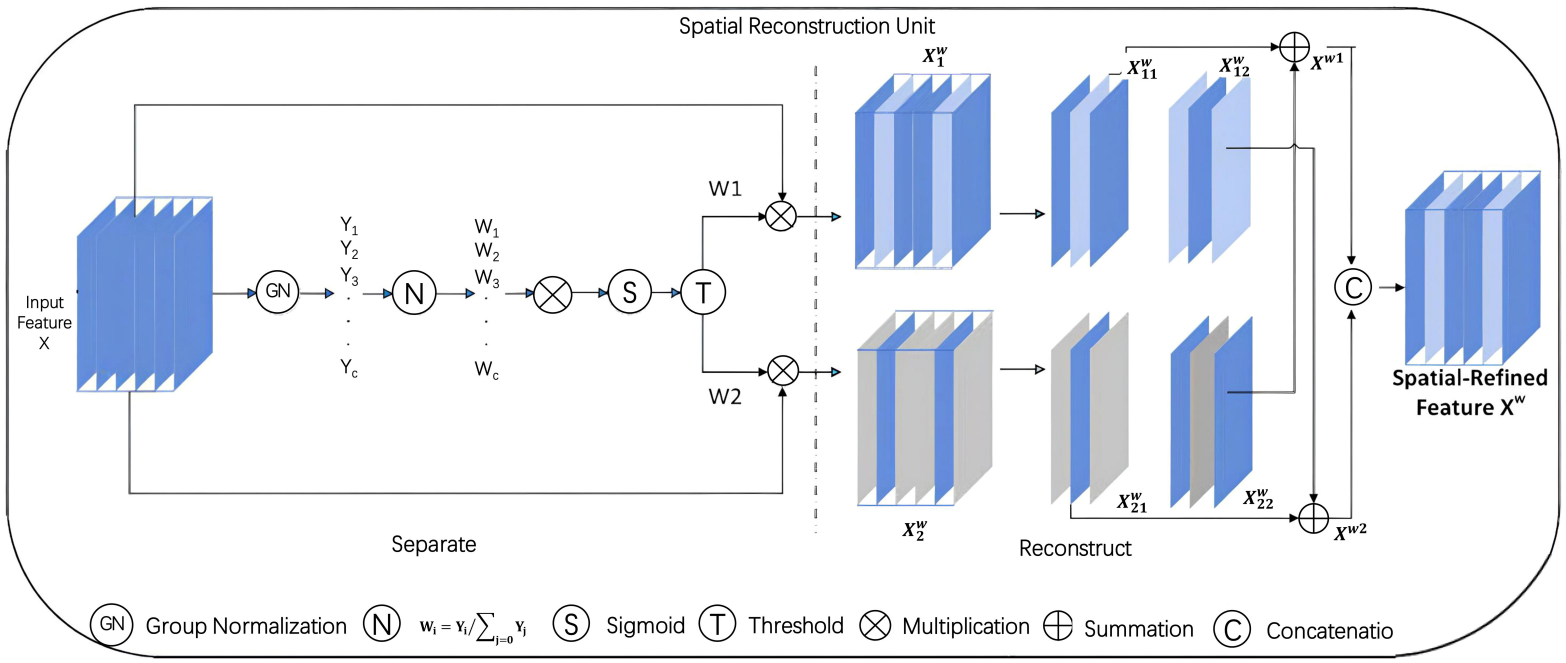
Space reconstruction unit.

**Figure 8:**
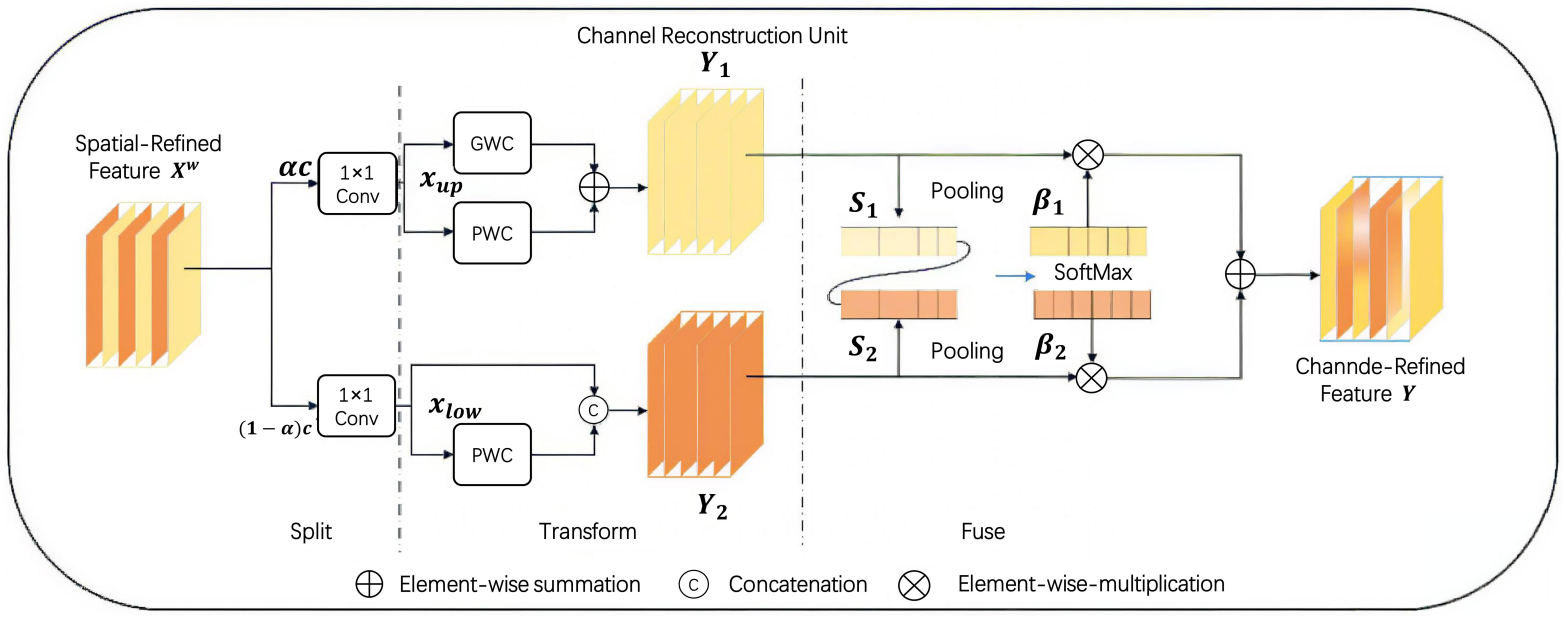
Channel reconstruction unit.

Consequently, in this study, a ScConv convolutional layer is incorporated into the Backbone network to boost both the network depth and performance. As depicted in **Figures 7**, **8** respectively, ScConv consists of two components: the Spatial Reconstruction Unit (SRU) and the Channel Reconstruction Unit (CRU).

The SRU adopts a separation - reconstruction method to effectively suppress spatially redundant features. Specifically, it decomposes the input feature map into multiple sub - feature maps, processes them separately, and then reconstructs the output. This approach can capture local spatial information more accurately and eliminate redundant spatial components.

The CRU, on the other hand, uses a segmentation - transformation - fusion strategy. It first divides the input channels into several groups, applies different transformations to each group, and then fuses the transformed channels. This way, it can successfully reduce channel redundancy and enhance the representational power of the channels.

This design, which strategically introduces the ScConv convolution at the bottom layer of the Backbone network, not only deepens the network but also optimizes its performance while keeping the number of parameters in check. As a result, it strikes a balance between the model’s accuracy and complexity, enabling more efficient and accurate processing in deep - learning tasks.

### 2.4 Method for measuring germination potential

In this study, a novel method is proposed with the aim of measuring the germination potential of seeds. The core concept of this method is to utilize a trained model to identify germinating seeds and then conduct research on their germination potential based on this identification. In this paper, the germination potential is defined as the germination rate, and the degree of change is characterized by the deformation of the seed during the germination process.

In the implementation of this method, the first step involves binarizing the images of germinating seeds into black - and - white images, where black represents the background and white represents the seed. Subsequently, the number of white pixel points is counted. This count serves to approximate the morphological changes that occur during seed germination. These data are then fitted to an equation that depicts the curve of change during germination. Through a process of derivation, a new equation is obtained, which represents the rate of change, namely, the germination potential as defined in this paper.

By employing this method, the germination potential of seeds can be effectively quantified, thereby offering theoretical support for subsequent research.

The specific operational steps are as follows: First, utilize a labeled dataset file in XML format to identify the target labeled as a germinating seed within it and extract its coordinates. Then, crop the germinating seed according to these coordinates to acquire an image containing only that particular seed.

Subsequently, perform black - and - white binarization on the image. After parameterization, a threshold value of 120 is set. Pixels with values less than 120 are classified as black, representing the background, while pixels with values greater than 120 are classified as white, representing the target seed, which includes the seed itself and its outgrowth parts.

Next, traverse the binary image and count and record the number of white pixel points. Finally, through mathematical modeling, fit the obtained data to a regression equation. After deriving this equation, a new equation is obtained for plotting a curve, which is used to describe the germination potential of the seeds. The pseudocode is presented as follows (i.e., **Algorithm 1**).

Algorithm 1 **Algorithm for measuring germination potential.**

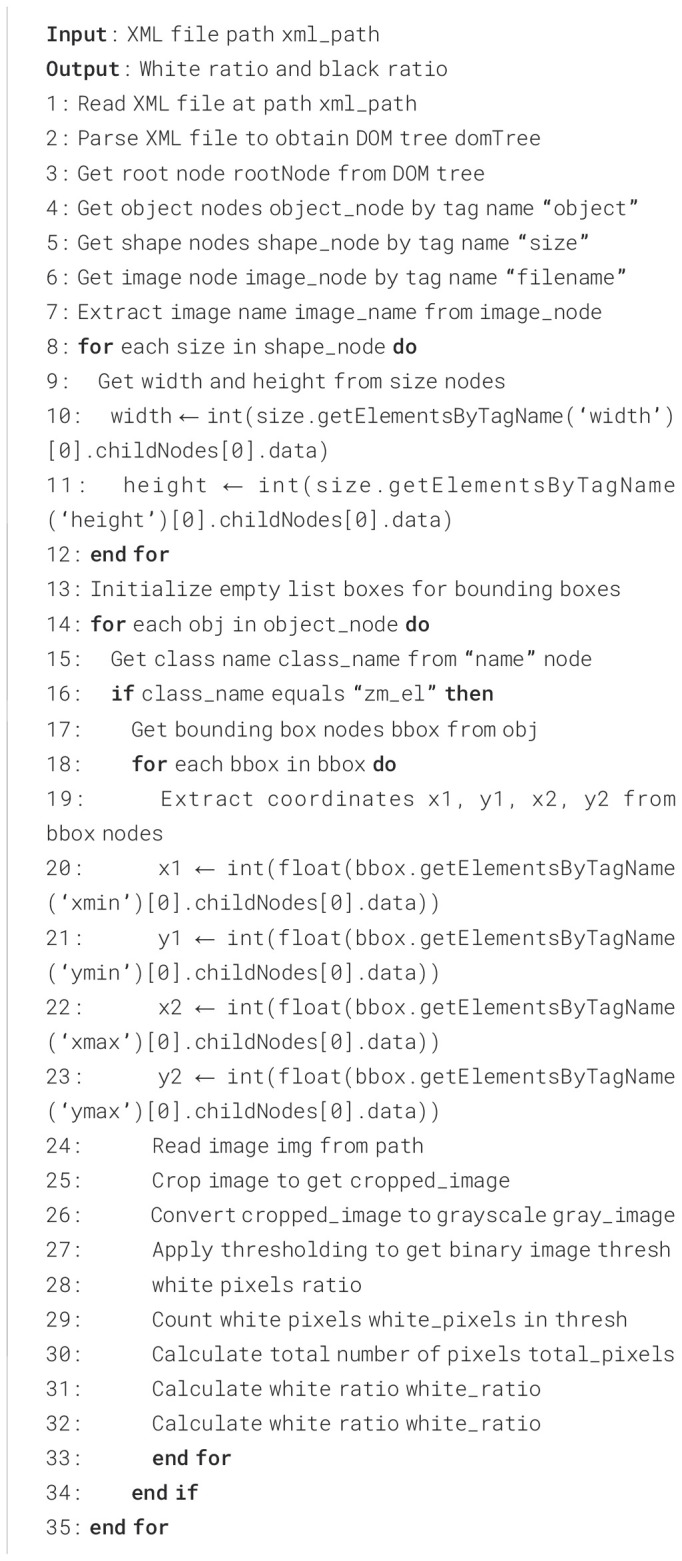

### 2.5 Experiment environments

In this experiment, the server’s runtime environment consists of the Pytorch deep - learning framework, operating on the Windows 10 Professional system. The system is equipped with an Intel(R) Xeon(R) W - 2245 CPU running at 3.90GHz, an NVIDIA Quadro RTX5000 GPU, and 64GB of RAM. It operates on CUDA 12.0 and utilizes libraries such as OpenCV to implement model training and subsequent measurements of the germination potential.

### 2.6 Evaluation indicators

In this experiment, when assessing the accuracy of the model regarding the germination status of corn seeds, the primary metrics employed are precision (Precision, Equation 1), recall (Recall, Equation 2), and mean average precision (mAP, Equations 3, 4). For the evaluation of the fitted regression equation, this paper utilizes the coefficient of determination (*R^2^*, Equation 5) as the assessment criterion. The relevant calculation formulas are presented as follows:


(1) $$Precision=\frac{TP}{TP+FP}\times 100\%$$

(2) $$Recall=\frac{TP}{TP+FN}\times 100\%$$

(3) $$AP=\int_{0}^{1}P(r)dr\times 100\%$$

(4) $$mAP=\frac{\sum_{1}^{N}\int_{0}^{1}P(r)dr}{N}\times 100\%$$

(5) $$R^{2}=\frac{SSE}{SST}=\frac{\sum_{i=1}^{N}{\left({\hat{y}}_{i}-\bar{y}\right)}^{2}}{\sum_{i=1}^{N}{\left(y_{i}-\bar{y}\right)}^{2}}$$

Where TP (True Positive) refers to the correct detection frame, which means that the prediction frame matches the labeled frame accurately. FP (False Positive) refers to the false detection frame, i.e., the background is incorrectly predicted to be an instance of the target object. FN (False Negative) denotes the missed detection frame, i.e., the model fails to detect a target object that should have been recognized. mAP (mean Average Precision) is used to assess the overall performance of the model, where AP refers to the detection precision of a single category and mAP is the average precision of multiple categories. SSE stands for explained sum of squares, sst stands for total sum of squares, and r2 represents the model’s ability to explain the dependent variable, with values ranging from 0 to 1. The closer the value is to 1, the better the model fits the data. The better the fit of the model to the data.

## 3 Results

### 3.1 Training process

In the experiments described in this paper, consistent initial training parameter settings are applied for each individual experiment. The input image size is set to 640x640. The number of model training epochs is 100. The learning rate is configured as 0.01. The intersection - over - union (IoU) ratio is set at 0.7. The momentum is set to 0.937, the weight decay is 0.0005, and the batch size of the dataset used for each training iteration is 8. The detailed parameter values are presented in **Table 2**.

**Table 2:** Training parameter.

Parameter	Value
Input size pixels	640*640
Epochs	100
Learning rate	0.01
Weight decay	0.0005
Momentum	0.937
Batch size	8

**Figure 9:**
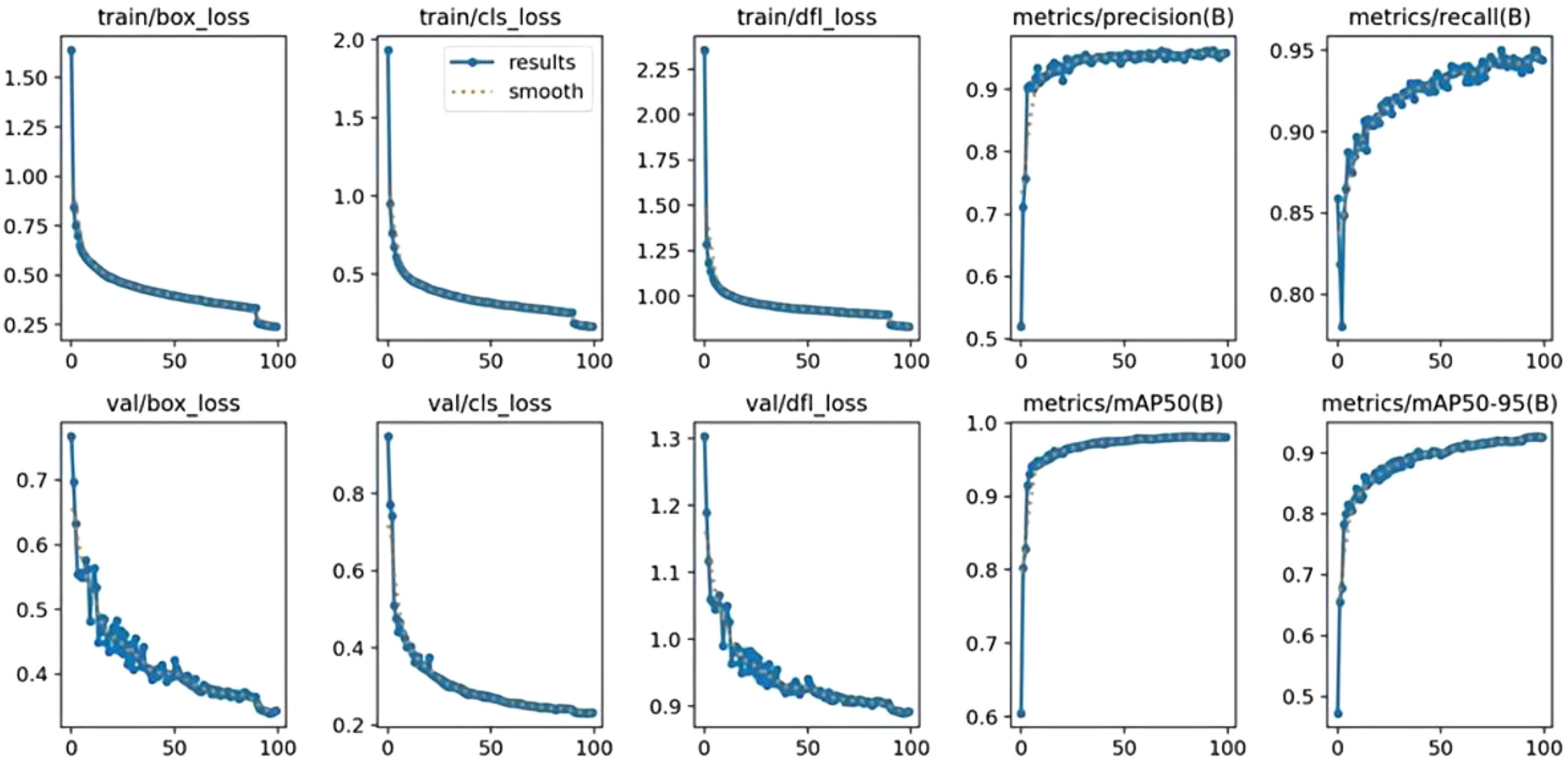
Model effect curve.

**Figure 9** illustrates that during the training process, the model’s training loss value varies according to the number of iterations, with the loss values gradually converging from the 30th iteration onwards. Regarding detection accuracy, it witnessed a substantial rise in the early stages, then started to improve slowly from the 10th iteration and stabilized after the 50th iteration. This trend indicates that the EBS - YOLOv8 model was trained without issues of overfitting and gradient vanishing, suggesting its effectiveness for the seed germination detection task.

### 3.2 Ablation experiment

In this study, mAP50 is defined as Mean Average Precision at IoU = 0.50, and mAP50-95 is defined as Mean Average Precision at IoU = 0.50:0.05:0.90.The first point of improvement was the introduction of the ECA attention mechanism, which improved the model’s accuracy by 0.7, recall by 1.2, mAP50 by 0.4, and mAP50-95 by 2.0 compared to the original model, while the number of parameters increased by only 0.17%. The second improvement point is the fusion of the small target detection layer with BiFPN, and this improvement results in an increase of 0.4 in accuracy, 1.8 in recall, 0.5 in mAP50, and 1.5 in mAP50-95 compared to the original model, while the number of parameters is reduced by 4.03%. The third point of improvement was the introduction of ScConv convolution, which showed an improvement of 0.2 in accuracy over the original model, 1.3 in recall, 0.3 in mAP50, 1.3 in mAP50-95, and a 3.98% increase in the number of parameters. Overall, all three improvement points effectively improve the base performance of the model without significantly increasing the number of parameters of the model, especially the second improvement point also successfully reduces the number of parameters of the base model. Next, we combine these three improvement points two by two to observe their impact on the results. The experimental results indicated that the mixing of two and two improved all the metrics compared to the original model. Finally, combining the three resulted in a 1.6 increase in accuracy, 2.3 increase in recall, 0.9 and 3.7 increase in mAP50 and mAP50-95, respectively, over the original model, while the number of parameters was reduced by 0.2% compared to the original model. This result indicated that the model performance was significantly improved without increasing the model complexity. The specific results are shown in **Table 3**.

**Table 3:** Ablation experiment.

ECA	P2BiFPN	ScConv	Precision(%)	Recall(%)	mAP50(%)	mAP50-95(%)	Param(s/M)
			95.1	94.0	98.0	92.1	3.01
✓			95.8	95.2	98.4	94.1	3.01
	✓		95.5	95.8	98.5	93.6	**2.88**
		✓	95.3	95.3	98.3	93.4	3.12
✓	✓		95.5	96.0	98.7	94.6	**2.88**
✓		✓	96.4	94.9	98.6	94.4	3.12
	✓	✓	95.7	94.8	98.4	94.1	3.00
✓	✓	✓	**96.7**	**96.3**	**98.9**	**95.8**	2.99

Key metrics with outstanding performance are highlighted in bold.

### 3.3 Comparison experiments

To further demonstrate the superiority of the EBS - YOLOv8 model presented in this paper, we conducted a comparison between the EBS - YOLOv8 model and several well - known counterparts, including Faster RCNN - ResNet50, SSD - VGG, YOLOv5s, CenterNet - ResNet50, and YOLOv7. The test results are detailed in **Table 4**.

**Table 4:** Model comparison.

Model	Precision(%)	Recall(%)	mAP50(%)	mAP50-95(%)	Params(M)
YOLOv8n	95.1	94.0	98.0	92.1	3.0
Faster Rcnn-ResNet50	92.9	91.6	89.9	55.5	137.1
SSD-VGG	93.6	89.9	90.1	81.9	26.3
YOLOv5s	94.3	94.5	97.4	90.4	7.1
CenterNet-ResNet50	96.5	90.8	92.6	88.7	32.67
EBS-YOLOv8	**96.7**	**96.3**	**98.9**	**95.8**	**2.99**

Key metrics with outstanding performance are highlighted in bold.

As shown in the table, the EBS - YOLOv8 model excels across all evaluation metrics. Specifically, its mAP50 and mAP50 - 95 values reach 98.9 and 95.8 respectively, significantly outperforming the other models. Moreover, the EBS - YOLOv8 model has the lowest number of parameters (Params), with only 2.99. This parameter count is the smallest among all the models involved in the comparison.

These findings clearly indicate that the EBS - YOLOv8 model manages to maintain high - level detection performance while effectively reducing the model’s complexity.

### 3.4 Germination rate test results

**Figure 10:**
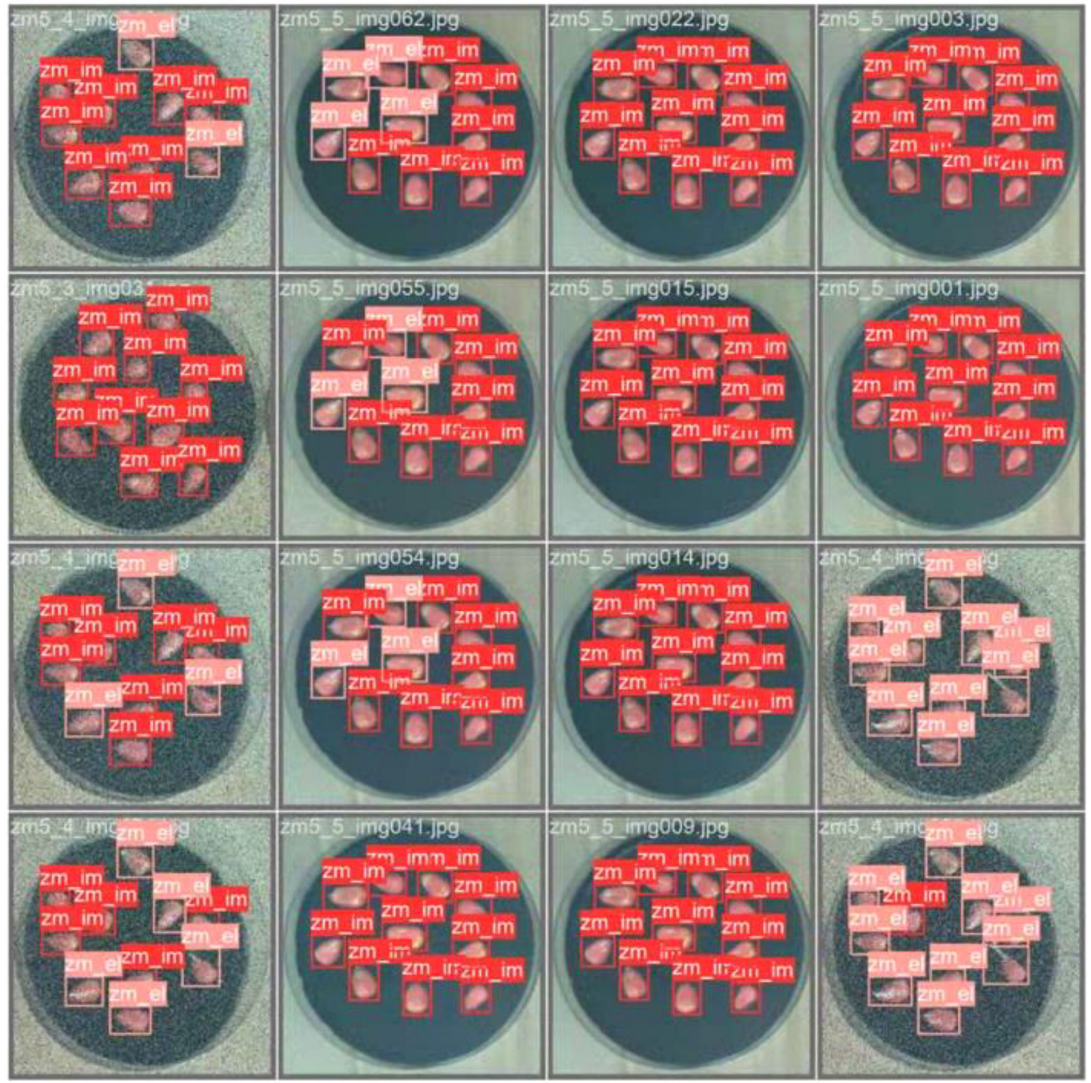
EBS-YOLOv8 model inspection chart.

To accurately evaluate the model’s performance, this paper selects images from the later germination stages of corn seeds for germination detection, with the relevant results presented in **Figure 10**. In the petri dish, as the corn seeds germinate, the buds exhibit overlapping and crossing, and their sizes vary significantly due to different growth rates. Despite these complex and diverse states, the EBS - YOLOv8 model demonstrates strong analytical capabilities, effectively identifying buds in various conditions.

In the experiment, a comparison was made between the results of manual observation and those of the EBS - YOLOv8 model’s detection on a test set consisting of 1223 sheets. Given that the germination - rate experiment aims solely to test the model’s effectiveness in detecting germinated seeds, in this experimental segment, this paper disregards the fact that some Petri dishes in the test set are duplicates and focuses on detecting the occurrence of germination. The obtained germination rate merely reflects the comparison between manual - observation and model - detection effects and does not represent the actual germination rate of the batch of seeds.

Manual observation was carried out throughout the germination cycle. To guarantee the experiment’s accuracy, three researchers independently observed the number of germinated maize seeds in the test set. For a total of 11,858 seeds, they finally recorded an average of 2,820 germinations, yielding a calculated germination rate of 23.8%. In contrast, based on the same number of seeds, the EBS - YOLOv8 model detected 3018 germinations, achieving a germination rate of 25.5%.

Regarding the detection of germination in each image, the manual - observation time per image was approximately 2.5 seconds. The cumulative total observation time for 1223 images was around 3057.5 s. When using the EBS - YOLOv8 model for seed - germination detection, in marked contrast, it significantly cuts down the time cost. With an average inference time of only 0.045 s per image, the total time amounts to 55.03 s, thus remarkably enhancing the detection efficiency. The detailed results are presented in **Table 5**.

**Table 5:** Statistical table of manual and modeled tests for germination.

Name	Number of germination	Germination rate(%)	Detection time(s)
**Artificial observation**	2820	23.8	3057.5
**Model detection**	3018	25.5	30.8

Key metrics with outstanding performance are highlighted in bold.

### 3.5 Germination test results

**Figure 11:**
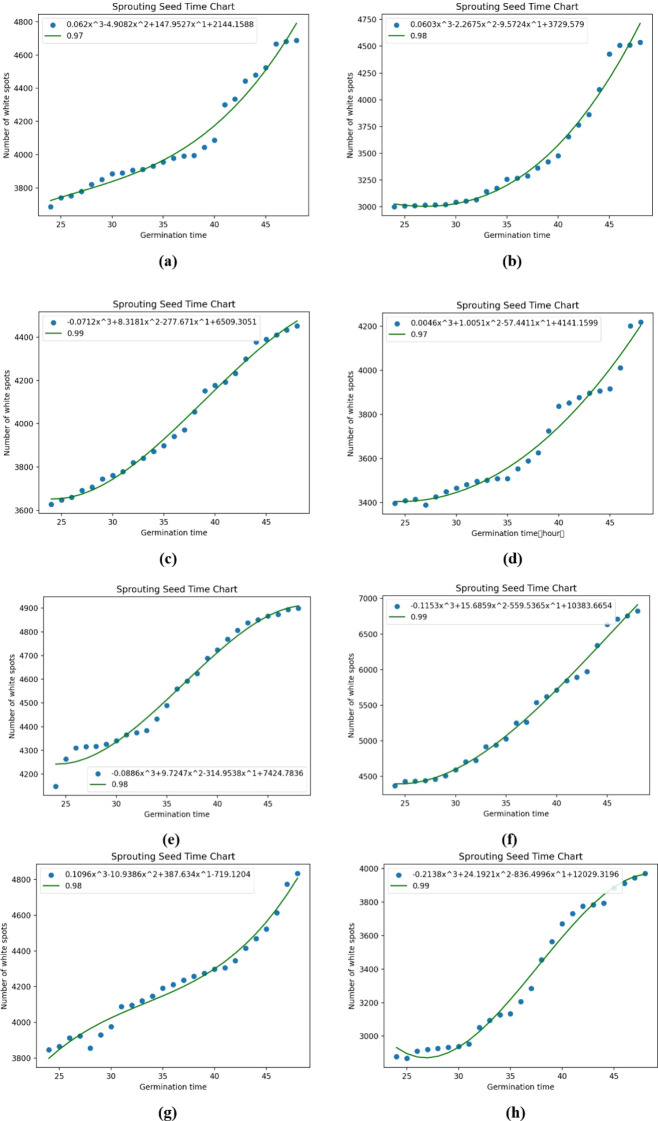
Numbers a-h represent the fitted equation curves for Petri dishes 1-8, respectively.

In this study, the germination potential is defined as the germination rate, different from the traditional definition which is the number of germinations within three days. In this paper, eight Petri dishes were randomly chosen and labeled as 1 - 8. By analyzing the bounding box coordinates output from the germinating seeds detected by the model, the best - performing germinating seeds are manually located through observation, and their time - series germination images are obtained for subsequent relevant research. After this series of binarization processes, the white portion in the image represents the seeds, while the black portion represents the background. Given that the main change in seeds during germination is the growth of buds, in this experiment, the number of white pixel points in the image was counted to objectively mirror the dynamic changes of germination. It was noted that the majority of seeds began to germinate 24 hours after the commencement of the germination experiment. Thus, 24 hours was set as the starting point, and the values of white pixel points at each time point were counted at 1 - hour intervals, followed by an analysis of the relationships among these data. In mathematical modeling, there are many methods for data fitting. Interpolation can be used to fit curves for function approximation, and a curve that meets the requirements is determined through a given set of data. However, the fitted curve will pass through all the given points, which fails to achieve our goal of observing the germination potential. This study aims to obtain a relatively simple approximation of the function by reflecting the overall changing dynamics of the data. Therefore, this study chose curve fitting to fit the regression equation. Among various equations, including exponential functions, linear functions, logarithmic functions, power functions, and polynomial functions, it was found that the polynomial function had the best fitting effect with the highest R² coefficient, as shown in **Table 6**. During the polynomial fitting process, based on the principles of mathematical modeling, generally, the higher the polynomial order, the higher the fitting accuracy tends to be initially, but it will reach a plateau after attaining the maximum value. This study revealed that the fitting accuracy of a third - order polynomial is superior to that of a second - order polynomial. Although the accuracy of a fourth - order polynomial is slightly higher than that of a third - order polynomial, when attempting to plot the curves for fourth - order and higher - order polynomials, it was observed that the fitted curves exhibited a decreasing trend, which is inconsistent with the experimental expectations. Therefore, in this experimental segment, third - order polynomial regression equations among the polynomials were selected for fitting, as depicted in **Figures 11a-h**, corresponding to Petri dishes 1 - 8 respectively.

**Table 6:** Comparison results of data fitting equations.

Number	Equation type	R2	Expression
1	Exponential equation	0.91	3886.e^0.0099x^
	linear equation	0.90	41.265x+3556.8
	Logarithmic equation	0.65	309.17ln(x)+3375.9
	Power equation	0.67	-3428.4x^0.0752^
	Third-order polynomial equation	**0.97**	0.062x^3^-4.9082x^2^+147.9527x+2144.1588
2	Exponential equation	0.88	2704.9e^0.0186x^
	linear equation	0.84	67.196x + 2606.2
	Logarithmic equation	0.54	473.88ln(x) + 2380.3
	Power equation	0.59	2530.7x^0.1327^
	Third-order polynomial equation	**0.98**	0.0603x^3^-2.2675x^2^-9.5724x+3729.579
3	Exponential equation	0.91	3258.8e^0.0089x^
	linear equation	0.90	33.052x + 3236.5
	Logarithmic equation	0.62	242.29ln(x) + 3104
	Power equation	0.65	3141.3x^0.0656^
	Third-order polynomial equation	**0.97**	0.0046x^3^+1.0051x^2^-57.4411x+4141.1599
4	Exponential equation	0.98	3535.7e^0.0094x^
	linear equation	0.97	37.843x + 3513.4
	Logarithmic equation	0.76	294.47ln(x) + 3322.1
	Power equation	0.79	3366.4x^0.0739^
	Third-order polynomial equation	**0.99**	-0.0712x^3^+8.3181x^2^-277.671x+6509.3051
5	Exponential equation	0.97	4155e^0.0071x^
	linear equation	0.97	32.312x + 4141.9
	Logarithmic equation	0.80	259.44ln(x) + 3960
	Power equation	0.82	3988.7x^0.0573^
	Third-order polynomial equation	**0.98**	-0.0886x^3^+9.7247x^2^-314.9538x+7424.7836
6	Exponential equation	0.97	4061.1e^0.0204x^
	linear equation	0.96	110.65x + 3917.6
	Logarithmic equation	0.70	836.56ln(x) + 3415.2
	Power equation	0.76	3677.7x^0.1572^
	Third-order polynomial equation	**0.99**	-0.1153x^3^+15.6859x^2^-559.5365x+10383.6654
7	Exponential equation	0.95	3767.2e^0.0086x^
	linear equation	0.94	36.468x + 3746.1
	Logarithmic equation	0.73	284ln(x) + 3561.2
	Power equation	0.76	3601x^0.0675^
	Third-order polynomial equation	**0.98**	0.1096x^3^-10.9386x^2^+387.634x-719.1204
8	Exponential equation	0.96	2699.6e^0.016x^
	linear equation	0.95	53.944x + 2647.1
	Logarithmic equation	0.70	410.57ln(x) + 2395.8
	Power equation	0.75	2497.7x^0.1233^
	Third-order polynomial equation	**0.99**	-0.2138x^3^+24.1921x^2^-836.4996x+12029.3196

Key metrics with outstanding performance are highlighted in bold.

**Figure 12:**
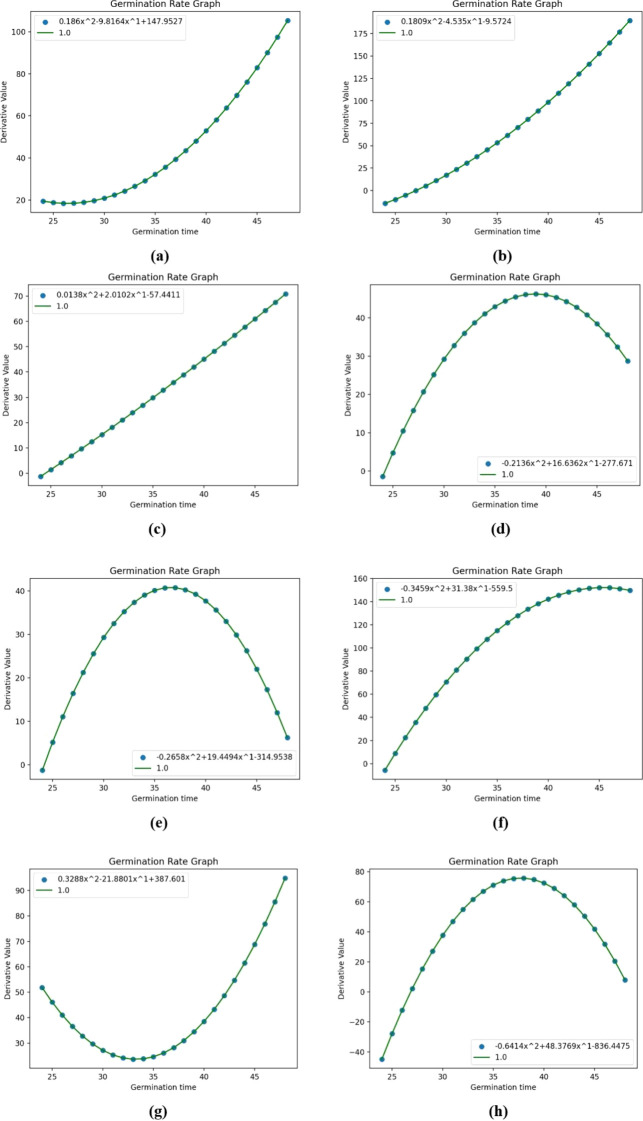
Numbers a-h correspond to Petri dish equation derivative curves 1-8, respectively.

In this study, the independent variable x of the one-dimensional cubic equation under consideration represents the change in time of germination in hours, while the dependent variable Y represents the increase in the number of white pixel dots of germinated seeds over time. This equation describes the change in the number of white pixel points of the seeds over time. To quantify the germination rate, the first-order derivative of the independent variable x of this equation was calculated to obtain a new equation whose derivative curve represents the change in germination potential, as shown in **Figure 12a-h**. In the figure, x is the time variation of germination, and Y is the derivative value corresponding to the value of x at that point, and the germination and derivative equations in each petri dish are shown in **Table 7**.

**Table 7:** Germination equation and derivative equation results.

Name	Curvilinear equation	R2	Derivative equation
1	0.062x^3^-4.9082x^2^+147.9527x+2144.1588	0.97	0.186x^2^-9.8164x+147.9527
2	0.0603x^3^-2.2675x^2^-9.5724x+3729.579	0.98	0.1809x^2^-4.535x-9.5724
3	0.0046x^3^+1.0051x^2^-57.4411x+4141.1599	0.97	0.0138x^2^+2.0102x-57.4411
4	-0.0712x^3^+8.3181x^2^-277.671x+6509.3051	0.99	-0.2136x^2^+16.6362x-277.671
5	-0.0886x^3^+9.7247x^2^-314.9538x+7424.7836	0.98	-0.2658x^2^+19.4494x-314.9538
6	-0.1153x^3^+15.6859x^2^-559.5365x+10383.6654	0.99	-0.3459x^2^+31.38x-559.5
7	0.1096x^3^-10.9386x^2^+387.634x-719.1204	0.98	0.3288x^2^-21.8801x+387.601
8	-0.2138x^3^+24.1921x^2^-836.4996x+12029.3196	0.99	-0.6414x^2^+48.3769x-836.4475

In summary, **Figure 11** depicts the temporal variation of seed morphology during the germination process. From a macroscopic perspective, by observing the curves, it is evident that the seed - morphology variable steadily increases throughout the entire germination process. Microscopically, the slopes of these curves mirror the magnitude of change at different time points, and this magnitude corresponds to the rate of change during various periods.

To conduct a more in - depth analysis of this process, the equation curve in **Figure 11** was differentiated, yielding a new equation curve presented in **Figure 12**. Mathematically, the derivative represents the slope. In this study, the graph of the derivative equation reflects the rate of change of germinating seeds over different germination times.

An analysis of **Figure 12** leads to the following conclusions: In Petri dish No. 1, the seed germination rate remained constant from 25 to 28 hours and then gradually rose after 28 hours. In Petri dish No. 2, the seed germination rate continuously increased starting from 25 hours. Petri dish No. 3 was similar to Petri dish No. 2, with a gradual upward trend. In Petri dishes Nos. 4, 5, and 8, the germination rates initially showed an increasing tendency, reaching their peak values at 38, 37, and 39 hours respectively, after which the rates began to decline gradually. Petri dish No. 6 exhibited a gradually increasing germination rate initially, which leveled off at 42 hours and then slightly decreased towards the end. Petri dish No. 7 had a relatively slow germination rate at the start, reaching its lowest point at 33 hours, after which the germination rate gradually increased.

## 4 Discussion

In summary, the EBS - YOLOv8 detection model put forward in this study demonstrated remarkable accuracy in detecting seed germination. The algorithm proposed for researching germination potential exhibits excellent feasibility and is capable of effectively characterizing the germination rate of seeds during the germination process. Consequently, this research furnishes a certain theoretical foundation and technical support for the issue of corn seed germination. Moreover, it offers novel perspectives for the study of the growth potential of other types of crop fruits. This is of substantial practical significance for the advancement of smart agriculture.

The dataset utilized in this study is composed of corn seed germination images captured over a 48 - hour span. This approach significantly cuts down the time cost in comparison to traditional 7 - day experiments. Although it deviates from the traditional 7 - day germination definition requirement, the changes in the seeds are highly conspicuous, and the emergence of germination is more pronounced. As a result, it offers abundant data support for training deep - learning models to detect seed germination.

After training, the proposed EBS - YOLOv8 detection model shows excellent performance on the test set, with an error rate of only 7% compared to actual manual observations. Through model improvement, the model’s adaptability in seed germination detection has been enhanced, demonstrating a substantial improvement over the original model. It can effectively identify germinated seeds. In contrast to traditional germination experiments, this method not only conserves human and material resources, reducing costs, but also enables non - destructive seed detection, thus increasing the reuse rate of experimental seeds.

The algorithm proposed in this paper for measuring germination potential uniformly classifies the seed itself and its germinating part as white pixel points. By analyzing the changes in these white pixel points and fitting the equation curve, it describes the deformation process of seed germination. Further derivation of the equation yields a new equation curve that can be used to depict the rate of seed germination deformation. Experimental results have proven the theoretical feasibility of this method, and the germination rate of seeds at different stages has been observed through curve analysis.

Compared with traditional germination potential studies, this research is more sensitive to seed quality. It is no longer restricted to simply counting the number of germinations but instead delves deeply into describing the germination trend and rate, which are more representative indicators. This study not only enables the measurement of the seed germination rate but also allows for the observation of seed germination potential, thereby providing theoretical support for seed selection and breeding.

**Figure 13:**
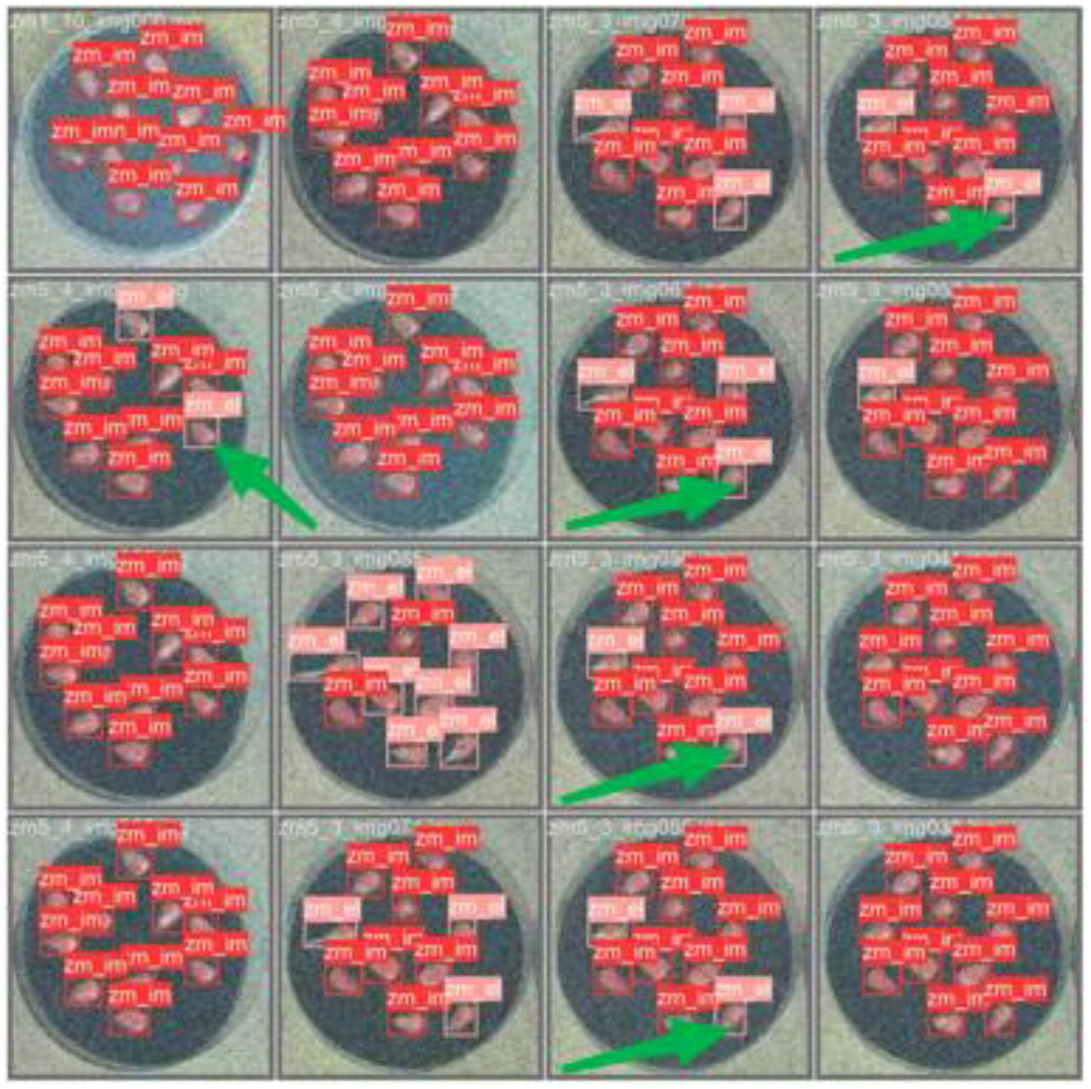
Model misdetection situation.

**Figure 14:**
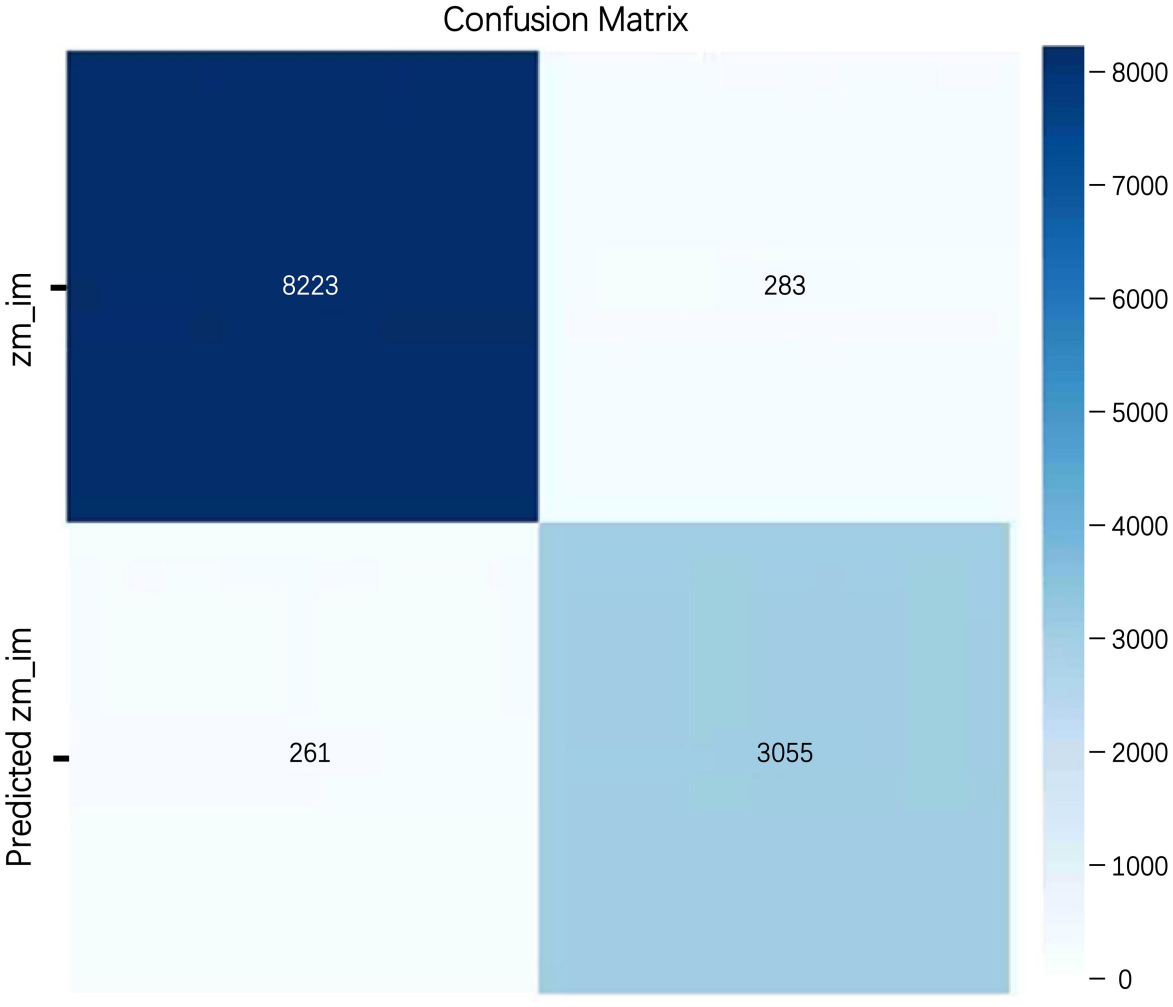
Confusion matrix.

However, this study has certain problems and limitations. The research on germination potential, which was based on the germination rate, revealed that some seeds were misclassified during germination detection, as illustrated in **Figure 13**. Analysis of the confusion matrix (**Figure 14**) showed that 11279 seeds were correctly classified during training, while 544 were misclassified. This phenomenon might be due to the dataset’s resolution issues and the fact that some non - germinated seeds at the time of detection had noise points misidentified as buds by the model, leading to misclassification as germinated seeds. Future research should further enhance the model’s ability to detect minute features to minimize the occurrence of false - detection phenomena.

**Figure 15:**
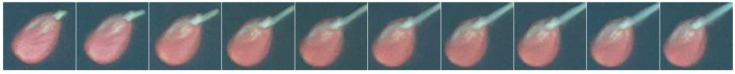
Seed time series germination images.

When conducting germination potential measurements, we noticed that for certain individual images, during the process of counting the white pixel points, a downward trend emerged. This led to negative values when calculating the derivative curve, a situation that runs counter to the actual biological principles. Through further in-depth investigation, we have identified that there are two primary causes contributing to this phenomenon:

One of the reasons is that the volume of individual seeds is likely to undergo changes during the germination process. Such changes may stem from the occurrence of drying out during germination, which in turn influences the statistical count of white pixel points. In the subsequent verification process, this assumption was corroborated by analyzing the image data, as depicted in **Figure 15**. The cropping dimensions utilized in the figure are uniform across all cases. It is evident that as time progresses, while the shoot part of the seed gradually grows, there is a concurrent tendency for the seed itself to shrink. This observed phenomenon indicates that fluctuations in seed volume can have a substantial impact on the measurement of germination potential, thereby affecting the accuracy of the model. Through an in-depth analysis of this phenomenon, we are of the opinion that future research endeavors should incorporate a quantitative analysis of seed volume variations. This approach is expected to enhance the accuracy and reliability of germination potential measurements.

Secondly, upon binarizing the images, we noticed that the morphology of certain seeds in consecutive germination images was inconsistent. This inconsistency might be attributed to the image’s own resolution and the conditions within the Petri dish. These factors can lead to edge defocusing and poor contour definition after binarization. Moreover, there are interference points surrounding the seeds that are imperceptible to the naked eye. These interference points may originate from impurities in the culture solution or dust particles that enter when the Petri dish lid is opened, ultimately influencing the experimental results.

**Figure 16:**
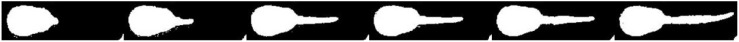
Binarised time series germination images.

We carried out experimental adjustments on multiple binarization methods and discovered that these issues were widespread, and they also had an impact on the fitting curves of our statistical data, as presented in **Figure 16**. This phenomenon indicates that optimizing the image - processing algorithm to enhance the capacity to detect the minute features of seeds will be beneficial in reducing errors and, consequently, improving the model’s ability to identify the germination status.

Therefore, the experimental results of germination potential in this study primarily provide an approximate depiction of the seed germination process, and in future research, the key problem to be resolved is how to describe the seed germination rate more precisely and rigorously on the existing foundation; the root cause of this problem is that in the current study, the seed body and the shoot body are regarded as an entirety, which can characterize the seed germination potential yet still has a certain error, so future research efforts should center on separating the seed body from the shoot body to carry out more accurate subsequent calculations, and with this enhancement, it is expected that the accuracy of germination potential measurements will be improved, thereby offering more reliable data support for the study of mechanisms related to plant growth.

## 5 Conclusion

Based on the YOLOv8n model, this paper presents an enhanced target - detection model, EBS - YOLOv8. This model is capable of effectively detecting germinating seeds, thereby providing crucial technical support for calculating seed germination rates in agricultural production and demonstrating great potential for real - time applications.

To achieve this, the ECA attention mechanism is incorporated to boost feature - extraction capabilities while maintaining the model’s lightweight nature. A small - target detection layer is added and upgraded to a BiFPN network, which significantly improves the detection accuracy of seed buds in the early germination stage. The ScConv convolution is applied to increase network depth, enhancing feature - extraction capabilities and optimizing model complexity. Moreover, the concept of “germination potential” is redefined and integrated with algorithms and mathematical - modeling techniques to enable visual measurement of the seed germination rate. These innovative measures substantially enhance the model’s performance and practical application value in seed - germination testing.

Through experimental verification, the EBS - YOLOv8 model exhibits excellent performance in seed - germination detection and can fully meet the requirements for measuring germination rates in agricultural production. This study holds significant application value in improving labor cost - effectiveness, production quality, and agricultural productivity. Additionally, by reducing computational resources and time consumption, it enhances the efficiency of practical applications, thus being of great practical significance for the promotion and utilization of target - detection technology.

## Data Availability

The data analyzed in this study is subject to the following licenses/restrictions: Available on request. Requests to access these datasets should be directed to zjy19970723@163.com.
